# The effects of family, society and national policy support on treatment adherence among newly diagnosed tuberculosis patients: a cross-sectional study

**DOI:** 10.1186/s12879-020-05354-3

**Published:** 2020-08-24

**Authors:** Xu Chen, Liang Du, Ruiheng Wu, Jia Xu, Haoqiang Ji, Yu Zhang, Xuexue Zhu, Ling Zhou

**Affiliations:** School of Public Health, Dalian Medical University, Dalian, 116044 Liaoning China

**Keywords:** Tuberculosis, Adherence, Family, Society, National policy

## Abstract

**Background:**

Non-adherence to tuberculosis (TB) treatment is the most important cause of poor TB outcomes, and improving support for TB patients is a primary priority for governments, but there has been little research on the effects of family, social and national policy support factors on TB treatment adherence. The current study evaluated treatment adherence among newly diagnosed TB patients in Dalian, north-eastern China, and determined the effects of family, society, and national policy support factors on treatment adherence.

**Methods:**

A cross-sectional survey was conducted among newly diagnosed TB patients treated at the outpatient department of Dalian Tuberculosis Hospital from September 2019 to January 2020. Data were collected using a questionnaire that measured medication adherence, family support, social support, and national policy support and so on. Differences between groups were assessed using Chi-square tests and Fisher’s exact tests. Ordinal logistic regression analysis was used to determine the predictors of adherence.

**Results:**

A total of 481 newly diagnosed TB patients were recruited, of whom 45.7% had good adherence, and 27.4 and 26.8% had moderate and low adherence, respectively. Patients who had family members who frequently supervised medication (*OR*:0.34, 95% *CI*:0.16–0.70), family members who often provided spiritual encouragement (*OR*:0.13, 95% *CI*:0.02–0.72), a good doctor-patient relationship (*OR*:0.61, 95% *CI*:0.40–0.93), more TB-related knowledge (*OR*:0.49, 95% *CI*:0.33–0.72) and a high need for TB treatment policy support (*OR*:0.38, 95% *CI*:0.22–0.66) had satisfactory medication adherence. However, patients who had a college degree or higher (*OR*:1.69, 95% *CI*:1.04–2.74) and who suffered adverse drug reactions (*OR*:1.45, 95% *CI*:1.00–2.11) were more likely to have lower adherence.

**Conclusions:**

Our findings suggested that non-adherence was high in newly diagnosed TB patients. Patients who had family members who frequently supervised medication and provided spiritual encouragement and a good doctor-patient relationship and TB-related knowledge and a high need for policy support contributed to high adherence. It is recommended to strengthen medical staff training and patient and family health education and to increase financial support for improving adherence.

## Background

Tuberculosis (TB), an infectious chronic respiratory disease caused by *Mycobacterium tuberculosis* complex (MTBC) has affected humans for thousands of years, and it is one of the top ten causes of death in the world [1]. In 2018, there were an estimated 10 million new cases of TB and 1.5 million deaths worldwide [2]. Approximately 1.7 billion people worldwide were infected with *Mycobacterium tuberculosis*, all of whom were at risk of developing TB [3]. China, one of the most heavily burdened countries in the world, still suffer from a severe TB epidemic. In 2018, there were 866,000 new TB patients, or 61 per 100,000 people [2], and the incidence has remained at a high level.

Why is the TB epidemic so difficult to control? The national government has always attached great importance to the prevention and control of TB and raised funding and implemented partial relief policies for TB. However, TB is currently treated with anti-TB medications, the treatment cycle of the disease is long, and adverse drug reactions are serious [4]. The symptoms of patients in the continuation phase are not significantly improved, and many patients also suffer considerable psychological, social and economic costs [5]. These issues lead to decreased patient adherence and irregular medication use. Several previous studies have shown that irregular treatment, i.e., poor patient adherence, is the most important reason for poor TB outcomes [6–8]. Low adherence will lead to treatment failure or even relapse into drug-resistant TB in patients with a smear-negative smear, which also leads to prolonged infectious periods in patients with a smear-positive smear and increase the number of drug-resistant and relapse cases [4, 9]. Other studies have also shown that non-adherence is the most common cause of relapse, and the relapse rate of patients with poor adherence is as high as 50.5%, whereas the relapse rate of patients with good adherence is only 1.1% [10]. For TB treatment, adherence is critical to achieving the desired treatment success rate [11]. Regular drug intake as prescribed is key to achieving a cure and avoiding the development of drug resistance while also protecting communities from the spread of TB [12]. Therefore, a study on the factors influencing adherence among newly treated TB patients and the discovery of new ways to improve adherence, not only contributes to improving TB cure rates but also to preventing the relapse of TB and the emergence of drug-resistant TB, which is crucial to the global goal of ending TB.

To gain a thorough understanding of the barriers and enablers of adherence, many countries have studied the factors that influence adherence. A study from Sri Lanka identified barriers to adherence related to patient and health service delivery [13]. Studies in Northwestern Ethiopia had shown that knowledge of disease and treatment, complications, alcohol abuse, forgetfulness and busy work schedules were significantly associated with non-adherence [14]. A cross-sectional study conducted in four regions of Russia confirmed that psychosocial factors also influenced adherence [15]. China is a country with a high TB burden that accounts for 9% of the world’s cases [2]. Studies on factors that influence adherence have been conducted in Anhui, Shenzhen, Hubei and other places. Studies have identified the relationship between medication adherence and socio-demographic characteristics, socio-economic factors, health systems and drug adherence [9, 16]. In addition, some studies have shown that psychological factors, such as stigma and depressive symptoms, significantly affect patient adherence [17]. Improving support for TB patients is a major priority for governments [18]. However, few studies have examined the impact of family, society and national policy support factors on TB treatment adherence.

In recent years, more studies have been conducted on the adherence of TB patients in China. However, most studies have focused on the adherence of migrant populations, patients from impoverished mountainous area, relapsed TB patients and multidrug-resistant TB patients. Few studies have been conducted only among newly diagnosed TB patients. Liaoning province is more prosperous than other provinces in the northeast of China. TB morbidity and mortality in the whole province occupied the first two places among infectious diseases. In 2018, the reported incidence of TB was 55.81/100,000, and the mortality rate was 0.49/100,000 in Liaoning province [19]. TB seriously endangers people’s health and affects the development of the economy and society in this province. Dalian is the best economically developed area in Liaoning province. In 2016, the incidence of TB was 64.66/100,000 in the general population of Dalian [20]. In recent years, although the incidence of TB has been declining slowly, it is still the main killer among infectious diseases.

### Aim

The aim of this study was to evaluate the level of adherence to anti-TB treatment among newly treated TB patients in Dalian, Liaoning province, Northeastern China, and we would further identify the potential effects of family, society and national policy support factors on adherence.

## Methods

### Study design and setting

A cross-sectional, questionnaire-based survey was conducted at the Dalian Tuberculosis Hospital in Liaoning province, north-eastern China from September 2019 to January 2020. Dalian Tuberculosis Hospital is the only TB control hospital in Dalian and is divided into two parts: the northern branch and the southern one. The hospital occupies an area of 45,000 square metres. It is responsible for the diagnosis, treatment, and prevention of and training and scientific research on TB and pulmonary diseases in Dalian. In 1994, an internationally recommended strategy for TB control, Directly Observed Treatment, Short-course (DOTS), was launched by the World Health Organization (WHO). In 2001, China started to implement the DOTS strategy comprehensively. To date, 100% supply is available in all counties (districts) of Liaoning Province. In addition, free chest radiographs and sputum smear tests are provided for patients with suspected symptoms of TB, such as cough, expectorant or bloody sputum. Newly diagnosed TB cases must be registered at the local TB dispensary and reported to upper level health authorities. To enhance treatment adherence, reduce interruptions in treatment and improve the completion rate of treatment of newly registered TB patients, Dalian municipal government has introduced a policy to benefit the people. Since January 1, 2011, transportation and nutrition subsidies have been provided to newly registered pulmonary TB patients (excluding tuberculous pleurisy and other extrapulmonary TB) and chronic prolonged pulmonary TB with positive sputum smear tests during the current year who were treated on time in our hospital. The subsidy standard is 45 yuan for a transportation allowance and 200 yuan for a nutrition allowance per month. The newly diagnosed patients are given the subsides for 6 months, and the patients who relapse receive it for 8 months.

### Participants

The study participants were made up of TB patients who met the inclusion criteria and had outpatient visits at the Dalian Tuberculosis Hospital from September 2019 to January 2020. The inclusion criteria included the following: (1) newly diagnosed TB patients, that is, the patient’s medical records indicate that the patient denied any prior anti-TB treatment or any history of anti-TB treatment beyond 30 days [21] (2) age is greater than or equal to 18 years, (3) patients who have begun to take anti-TB drugs and have no mental disorder, (4) patients who easily communicate and can understand the contents of the questionnaire, and (5) patients who voluntarily agree to participate in this study and can truly express their views on the problem. A total of 485 patients were interviewed in this study, of which 4 did not complete the interview, and the response rate was 99.2%. In the end, the study included 481 patients.

### Data collection

Structured questionnaires were used for data collection. The questionnaire was designed after we consulted a large amount of relevant literature at home and abroad and consulted experts in related fields. In addition, a preliminary survey was conducted at the survey site, and the questionnaire was supplemented and modified according to the results of the preliminary survey to ensure the validity of the questionnaire. The questionnaire was composed of medication adherence, socio-demographic characteristics, adverse drug reactions, family support, social support and national policy support factors. The data were collected by a team of trained and qualified graduate students.

The adherence of TB patients was investigated using the eight-item Morisky Medication Adherence Scale (MMAS-8) [22]. This scale is one of the simplest ways to measure a patient’s medication adherence. The scale consists of eight items that measure a specific medication behaviour. The highest score on this scale was 8, indicating high adherence. A score less than 8 but not less than 6 was considered moderate adherence, and a score less than 6 was classified low adherence [23, 24]. This scale had good reliability and sensitivity, and the Cronbach’s α was 0.81 in this study.

The sociodemographic characteristics measured in this study included 6 questions involving gender, age, marital status, education level, self-reported average monthly income, and the time required to arrive at a health facility. The family support section consisted of 4 questions, including supervision of medication, spiritual encouragement, the relationship between family members, and help to solve problems in daily life. Social support was made up of 11 questions covering care, support and help from friends, neighbours, colleagues and doctors and the patient’s participation in group activities and acquired knowledge of TB. Patients’ access to TB knowledge was assessed by asking 6 questions, such as how TB is transmitted and how to treat it. Patients got one point for each correct answer, and higher scores indicate better understanding. In this study, 2 points and 4 points were used as segmentation points to divide knowledge into three levels. The national policy support module was composed of 4 issues: policy understanding, policy satisfaction and the need for increased national TB treatment policies. The last question in the module was an open question on what policy support was still needed (See Additional file 1).

### Data analysis

On the day the questionnaires were returned, researchers checked the completeness of questionnaires and eliminated illogical data. After ensuring that the questionnaire was complete and correct, the questionnaire was coded. Data were entered into the database established by software EpiData3.1 (EpiData Association, Odense, Denmark) using the double-entry method. The results were tested for consistency, and the original questionnaires were searched for inconsistencies. An Excel database was established, and SPSS 21.0 (IBM Corporation, Armonk, State of New York) was used for statistical analysis. Quantitative data were described by means and standard deviations. Categorical data were described by frequency and percentage. Chi-square tests were used to evaluate differences in the categorical data between different groups. For scarce data, we used Fisher’s exact tests. All statistically significant variables in the univariate analyses were entered into ordinal logistic regression analysis to determine the predictors of adherence. Odds ratios (*ORs*) and their 95% confidence intervals (*CIs*) were calculated. If *P* < 0.05, the difference was statistically significant.

## Results

### The level of non-adherence to anti-TB treatment

A total of 481 newly diagnosed TB patients were included in this study. The mean score of the medication adherence scale was 6.53 ± 1.85. In all, 220 (45.7%) patients included in our study were classified as having good adherence, and 132 (27.4%) and 129 (26.8%) had moderate and low adherence, respectively.

### Socio-demographic characteristics and adverse drug reactions

In our study, patients ranged in age from 18 to 88 years, with an average age of 44.10 ± 17.85 years. More than half of the TB patients (62.0%) entering the study were male, whereas only 183(38%) were female. Nearly three-quarters of TB patients (71.5%) were married, compared with 18(3.7%) who were divorced or widowed. TB patients with an education level of secondary school or below (41.0%) accounted for the largest percentage. Most TB patients have low monthly incomes, with only 84 (17.5%) of TB patients earning more than 5000 yuan a month. Nearly one third of TB patients (32.0%) arrived at the facility in more than an hour, and 136 (28.3%) arrived in less than 30 min. Self-reported adverse reaction was mentioned from 184 (38.3%) of the participants. The results of the univariate analysis showed that education level, the time required to reach the medical institution and adverse drug reactions were significantly correlated with the medication adherence of TB patients (*P* < 0.05), and the adherence of TB patients of different genders, ages, marital status, and monthly income in our study were not different (*P* > 0.05). (Table 1).

**Table 1:** Medication adherence by sociodemographic factors and adverse drug reactions

Variables	Total n (%)	Adherence Level n (%)			*P*
		Low	Medium	High	
Sex					
Male	298 (62.0)	78 (26.2)	84 (28.2)	136 (45.6)	0.869
Female	183 (38.0)	51 (27.9)	48 (26.2)	84 (45.9)	
Age (years)					
< 30	128 (26.6)	36 (28.1)	37 (28.9)	55 (43.0)	0.942
30–60	244 (50.7)	65 (26.6)	67 (27.5)	112 (45.9)	
> 60	109 (22.7)	28 (25.7)	28 (25.7)	53 (48.6)	
Marital status					
Unmarried	119 (24.7)	35 (29.4)	42 (35.3)	42 (35.3)	0.096
Married	344 (71.5)	90 (26.2)	85 (24.7)	169 (49.1)	
Divorced or widowed	18 (3.7)	4 (22.2)	5 (27.8)	9 (50.0)	
Education					
Junior high school or below	197 (41.0)	52 (26.4)	54 (27.4)	91 (46.2)	**0.025**
High school or technical secondary school	117 (24.3)	29 (24.8)	22 (18.8)	66 (56.4)	
College degree or above	167 (34.7)	48 (28.7)	56 (33.5)	63 (37.7)	
Monthly income (Yuan)					
< 1000	133 (27.7)	31 (23.3)	42 (31.6)	60 (45.1)	0.210
1000–3000	115 (23.9)	34 (29.6)	33 (28.7)	48 (41.7)	
3001–5000	149 (31.0)	40 (26.8)	30 (20.1)	79 (53.0)	
> 5000	84 (17.5)	24 (28.6)	27 (32.1)	33 (39.3)	
Time to arrive at the medical facility (min)					
< 31	136 (28.3)	36 (26.5)	26 (19.1)	74 (54.4)	**0.033**
31–60	191 (39.7)	45 (23.6)	62 (32.5)	84 (44.0)	
> 60	154 (32.0)	48 (31.2)	44 (28.6)	62 (40.3)	
Adverse drug reactions					
Yes	184 (38.3)	64 (34.8)	49 (26.6)	71 (38.6)	**0.005**
No	297 (61.7)	65 (21.9)	83 (27.9)	149 (50.2)	

### Family support

Table 2 shows that the family supervising medication, family spiritual encouragement, and family member relationships were significantly different in different groups by univariate analysis (*P* < 0.05). A high proportion of low adherence (48.6%) was found in TB patients whose family members sometimes supervised their medication, whereas a high proportion of high adherence (47.7 and 54.5%, respectively) was found in the two groups of patients whose family members frequently supervised their medication and those who did not. Patients with frequent spiritual encouragement from family members had a higher proportion of high adherence (47.0%). The relationship between family members of most patients (95.6%) was good, and there was a small proportion of poor adherence (26.3%). A significantly higher proportion of patients (91.3%) had family members who were able to regularly help solve problems in daily life (Table 2).

**Table 2:** Medication adherence by family support factors

Variables	Total n (%)	Adherence Level n (%)			*P*
		Low	Medium	High	
Family supervision for medication					
Often	411 (85.4)	105 (25.5)	110 (26.8)	196 (47.7)	**0.002**
Sometimes	37 (7.7)	18 (48.6)	13 (35.1)	6 (16.2)	
Never	33 (6.9)	6 (18.2)	9 (27.3)	18 (54.5)	
Family spirit encouragement					
Often	451 (93.8)	116 (25.7)	123 (27.3)	212 (47.0)	**0.012**^a^
Sometimes	20 (4.2)	10 (50.0)	3 (15.0)	7 (35.0)	
Never	10 (2.1)	3 (30.0)	6 (60.0)	1 (10.0)	
Family relationship					
Good	460 (95.6)	121 (26.3)	124 (27.0)	215 (46.7)	**0.012**^a^
General	17 (3.5)	8 (47.1)	4 (23.5)	5 (29.4)	
Poor	4 (0.8)	0 (0.0)	4 (100.0)	0 (0.0)	
Family members help solve problems					
Often	439 (91.3)	112 (25.5)	122 (27.8)	205 (46.7)	0.134
Sometimes	24 (5.0)	12 (50.0)	5 (20.8)	7 (29.2)	
Never	18 (3.7)	5 (27.8)	5 (27.8)	8 (44.4)	

^a^Means that the theoretical number is too small, and the Fisher’s exact test was used

### Societal support

TB patients who had one or two close friends accounted for 46.6% of the patients included in the study, but 51 (10.6%) had no close or supportive friends. Nearly 30% of TB patients reported poor relationships with neighbours and co-workers (29.7 and 27.0%, respectively). More than half of TB patients (56.5%) reported that they never participated in group activities, whereas only 41 (8.5%) were regularly or actively involved in group activities. Patients generally had a good doctor-patient relationship, with a small minority (19.3%) reporting a poor relationship with the medical staff. The mean score for knowledge about TB in the included TB patients was 4.72 ± 1.20, with 146 (30.4%) getting full marks, but 24 (5%) of the patients still scored less than 3 points. Univariate analysis found that doctor-patient relationship, acquired knowledge of TB and participation in group activities were correlated with medication adherence among TB patients (*P* < 0.05). (Table 3).

**Table 3:** Medication adherence by society support factors

Variables	Total n (%)	Adherence Level n (%)			*P*
		Low	Medium	High	
The number of close friends					
0	51 (10.6)	17 (33.3)	12 (23.5)	22 (43.1)	0.729
1–2	223 (46.4)	62 (27.8)	61 (27.4)	100 (44.8)	
≥ 3	207 (43.0)	50 (24.2)	59 (28.5)	98 (47.3)	
Relationships with neighbours					
Poor	143 (29.7)	36 (25.2)	45 (31.5)	62 (43.4)	0.394
General	220 (45.7)	66 (30.0)	57 (25.9)	97 (44.1)	
Good	118 (24.5)	27 (22.9)	30 (25.4)	61 (51.7)	
Relationships with colleagues					
Poor	130 (27.0)	32 (24.6)	31 (23.8)	67 (51.5)	0.566
General	225 (46.8)	64 (28.4)	66 (29.3)	95 (42.2)	
Good	126 (26.2)	33 (26.2)	35 (27.8)	58 (46.0)	
Relationships with doctors					
Poor	93 (19.3)	30 (32.3)	29 (31.2)	34 (36.6)	**0.001**
General	223 (46.4)	66 (29.6)	69 (30.9)	88 (39.5)	
Good	165 (34.3)	33 (20.0)	34 (20.6)	98 (59.4)	
Acquired knowledge of TB					
Poor	24 (5.0)	7 (29.2)	7 (29.2)	10 (41.7)	**0.016**
General	149 (31.0)	55 (36.9)	37 (24.8)	57 (38.3)	
Good	308 (64.0)	67 (21.8)	88 (28.6)	153 (49.7)	
Participation in group activities					
Often	41 (8.5)	11 (26.8)	14 (34.1)	16 (39.0)	**0.008**
Sometimes	168 (34.9)	55 (32.7)	53 (31.5)	60 (35.7)	
Never	272 (56.5)	63 (23.2)	65 (23.9)	144 (52.9)	

### National policy support

The number of patients (29.3%) who knew about the country’s treatment policies for TB was relatively small. There was no significant association with medication adherence (*P* > 0.05), although patients who did not know had a higher percentage of low adherence. Satisfaction with the national medical security policy for TB treatment and the need to increase policy support for TB treatment were proved to be relevant factors (*P* < 0.05). The proportion of TB patients who were satisfied with the national medical security policy for TB treatment was 50.7%, whereas the proportion who were generally satisfied or less satisfied with the medical security policy was 39.3 and 10.0%, respectively. Most patients (96.7%) believed that the government still needed to increase support for TB treatment (Table 4).

**Table 4:** Medication adherence by national policy support factors

Variables	Total n (%)	Adherence Level n (%)			*P*
		Low	Medium	High	
National TB treatment policy					
Know	141 (29.3)	35 (24.8)	31 (22.0)	75 (53.2)	0.168
General	143 (29.7)	39 (27.3)	38 (26.6)	66 (46.2)	
Unknow	197 (41.0)	55 (27.9)	63 (32.0)	79 (40.1)	
Medical security policy satisfaction					
Satisfaction	244 (50.7)	66 (27.0)	52 (21.3)	126 (51.6)	**0.004**
General satisfaction	189 (39.3)	54 (28.6)	58 (30.7)	77 (40.7)	
Not too satisfaction	48 (10.0)	9 (18.8)	22 (45.8)	17 (35.4)	
Increase support for TB treatment policies					
Need	408 (84.8)	98 (24.0)	113 (27.7)	197 (48.3)	**0.004**
General need	57 (11.9)	27 (47.4)	15 (26.3)	15 (26.3)	
Not too need	16 (3.3)	4 (25.0)	4 (25.0)	8 (50.0)	

### Ordinal logistic regression analysis of factors independently associated with medication adherence among patients

Ordinal logistic regression analysis showed that patients whose family members regularly supervised medication (*OR*:0.34, 95% *CI*:0.16–0.70) and whose family members often encouraged them mentally (*OR*:0.13, 95% *CI*:0.02–0.72) were more likely to have a high medication adherence. Patients with a better doctor-patient relationship (*OR*:0.61, 95% *CI*:0.40–0.93) and more TB-related knowledge (*OR*:0.49, 95% *CI*:0.33–0.72) were less likely to have low medication adherence. Patients who want greater support for TB treatment policies (*OR*:0.38, 95% *CI*:0.22–0.66) were more likely to have good medication adherence. However, patients with a college degree or higher (*OR*:1.69, 95% *CI*:1.04–2.74) and those who had experienced adverse drug reactions (*OR*:1.45, 95% *CI*:1.00–2.11) were more likely to have low medication adherence (Table 5).

**Table 5:** Ordinal logistic regression analysis and predictors of medication adherence

Variables	*OR*	95% *CI*	*P*
Education (Ref: College degree or above)			
Junior high school or below	1.13	0.73–1.75	0.571
Technical secondary school or high school	1.69	1.04–2.74	**0.033**
Time to arrive at the medical facility (Ref:> 60 mins)			
< 31	1.19	0.74–1.89	0.477
31–60	1.17	0.77–1.77	0.474
Adverse drug reactions (Ref: Yes)			
No	1.45	1.00–2.11	**0.048**
Family supervision for medication (Ref: Often)			
Never	2.73	1.17–6.37	**0.020**
Sometimes	0.34	0.16–0.70	**0.004**
Family spirit encouragement (Ref: Often)			
Never	0.13	0.02–0.72	**0.019**
Sometimes	0.73	0.27–1.99	0.542
Family relationship (Ref: Good)			
Poor	3.05	0.26–35.90	0.375
General	0.95	0.34–2.63	0.919
Relationships with doctors (Ref: Good)			
Poor	0.75	0.43–1.28	0.289
General	0.61	0.40–0.93	**0.021**
Acquired knowledge of TB (Ref: Good)			
Poor	0.78	0.34–1.80	0.555
General	0.49	0.33–0.72	**< 0.001**
Participation in group activities (Ref: Often)			
Never	1.23	0.64–2.38	0.533
Sometimes	0.84	0.43–1.63	0.605
Medical security policy satisfaction (Ref: Satisfaction)			
Not too satisfaction	0.95	0.50–1.78	0.867
General satisfaction	0.86	0.58–1.26	0.426
Increase support for TB treatment policies (Ref: Need)			
Not too need	0.85	0.31–2.32	0.751
General need	0.38	0.22–0.66	**0.001**

*Ref* Reference

Variables with a *P*-value less than 0.05 in the univariate analysis were included in the ordinal logistic regression analysis. These variables were education, time to arrive at the medical facility, adverse drug reactions, family supervision for medication, family spirit encouragement, family relationship, relationships with doctors, acquired knowledge of TB, participation in group activities, medical security policy satisfaction and increase support for TB treatment policies.

### Patients’ advice for increased policy support in an open question

**Fig. 1:**
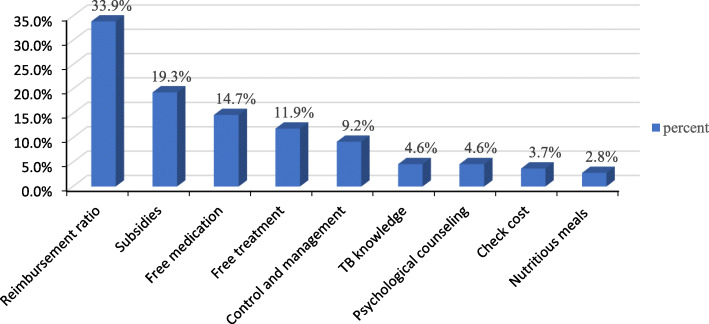
The percent of advice for more policy support for TB patients

Some TB patients stated one or more pieces of advice for greater policy support, and advice was received from 109 TB patients. More economic support (87.2%) was most frequently proposed, and included increased reimbursement rates (33.9%), increased subsidies for nutrition, transportation, and other expenses (19.3%), increased free drug coverage and time (14.7%), free treatment (11.9%), and reduced testing costs (3.7%), especially among poor patients and patients with first-lines drug resistance. The next most common advice was to increase TB prevention and patient management (especially isolation and education of infectious patients) (9.2%), to propagandize knowledge of TB (4.6%), to increase psychological counselling (4.6%), and to provide nutritious meals to inpatients or to improve the quality of food and beverages (2.8%).There are also a few patients who proposed to strengthen infrastructure construction, improve out-of-town reimbursement, provide job security and medication supply security for patients, optimize the problems of hospital transfer, reduce drug side effects, improve the success rate of treatment, and increase special protections for patients with comorbidities and students (Fig. 1).

## Discussion

As an infectious disease with high burden, the prevention and control of TB has attracted attention in China. Improving medication adherence in TB patients is considered an important way to effectively prevent and control TB. High-quality efficacy depends on patients’ adherence to the treatment regimen, and patient adherence is the key to successful treatment [25]. Not only does non-adherence pose a risk to the health of families and close contacts, it can also lead to the emergence of resistant bacteria that are more difficult and expensive to treat [26]. In our study, the percent of newly diagnosed TB patients with low adherence was 26.8%, whereas patients with good adherence were only 45.7% of participants. The proportion of low adherence was lower than that in the studies conducted in Shandong (34.6%) [27] and Hubei (33.3%) [17]. This difference may be related to the study design and the regional context. This finding also suggests that it is necessary to find an effective way to identify high-risk groups with non-adherence and measures to improve adherence.

The WHO reported that the largest burden of TB was in adult men [2], as in our study, where there were 1.63 times as many males as females. Gender was not associated with adherence, and similar results have been reported in other studies [28]. Most patients had a secondary or lower education level, and education level had an impact on adherence. Patients with a college degree or above made up a larger proportion of low adherence, which may be related to the living and working environment, and patients with a busy schedule tend to forget to take their medicine [14]. This issue may also be due to the fear that colleagues will know about their illness, and they do not regularly take medicine. However, the level of education is often associated with the patient’s knowledge of TB, which also has an impact on adherence [29]. Therefore, further investigation of this group will be needed in the future to obtain more evidence as a guide. It has been reported that time taken to reach a medical facility was associated with poor adherence, and we also found this phenomenon in univariate analysis [16]. Taking too long to get to the medical facility required higher transportation costs, and patients who were financially constrained by transportation costs had a higher risk of non-adherence to treatment [30, 31]. Some of the obstacles to treatment can be overcome by a reasonable increase in transport subsidies [32]. In addition, our study found that patients who experienced adverse drug reactions were more likely to be non-adherence. Adverse reactions caused by anti-TB drugs are a serious problem faced by TB patients [33]. If patients do not understand why adverse reactions occur, they are likely to develop severe anxiety about them. This issue may not only exacerbate the severity of adverse reactions but may also lead to non-adherence to treatment [34]. It is necessary to monitor and guide patients to ensure timely detection and management of adverse reactions during medication use.

The attitudes of family members may influence the patient’s decision to stop or continue treatment [35]. Family members, especially spouses, play an extremely important role in encouraging, supporting and supervising the patient’s medication [27]. Our study also found that patients with frequent medication supervision by family members and patients whose family members often encouraged them mentally were more likely to have a high level of adherence. This effect may be because TB patients generally carry a psychological burden of fear of treatment failure and a lack of confidence in curing the disease and hinder their adherence to treatment [36]. However, the constant encouragement and care of the family can increase the patient’s confidence, and thus, affect the patient’s medication adherence. During the illness, family members help solving problems in their lives had no effect on adherence. Some experts believed that when intervening in patient adherence, it was important whether the patient feels supported [37], whereas there was a lack of usual involvement in helping to solve problems. Our study also found that patients whose family members sometimes supervised medication were more likely to have poor adherence, possibly due to family members not supervising when the patient forgot to take medication. Directly observed therapy (DOT) is considered a timely reminder and can improve medication adherence [38]. Studies have shown that DOT is mostly provided by family members and that well-trained family members will provide better DOT than health services personnel [39]. With doctors having limited time and sometimes skill to repeatedly instruct patients to stick to their medications [40], it may make sense to transfer DOT to family members. Therefore, we can improve patient adherence by training family members to better provide therapeutic and psychological interventions. Univariate analysis found that the relationship between family members and adherence was also related. Close family relationships can increase patients’ life satisfaction, disencumber their mind from care, and enhance their ability to fight disease, and patients with family dysfunction are more likely to be alienated and lead to negative treatment [41]. That findings suggests that family members can not to be ignored in the patient’s treatment process.

In our study, the patients’ knowledge of TB was relatively good, with more than half getting full or near full marks, and patients who had a good knowledge of TB were more likely to show adherence. Many other studies have also shown that health education had a positive effect on adherence [42]. Patients’ lack of knowledge about TB often meant an incorrect understanding of TB [41]. Some patients also express a desire for knowledge and hope to popularize knowledge of TB. Therefore, it is necessary for hospitals to propagandize about prevention and treatment of TB and knowledge about TB to patients and their families before patients receive chemotherapy. Our research also showed that only doctor-patient relationships affected adherence rather than friends, colleagues and neighbours. The key to a good doctor-patient relationship is effective communication, and sometimes doctors fail to effectively explain the benefits and side effects of medications to patients and fail to fully consider the financial burden to patients, resulting in non-adherence [43]. This study was consistent with the findings of other studies, and more attention was also recommended to improve doctor-patient communication [44]. Home visits and telephone supervision contacts by health workers could improve treatment adherence, and health worker calls were a generally accepted method for patient management [45]. A study in Vietnam found that digital monitoring was also a viable and acceptable adherence support method [46]. However, there have also been studies that suggest that when patient adherence problems were reported to doctors through monitoring, doctors may not have enough financial incentives to manage patients more strictly [37]. Hence, improving doctor-patient relationships by training and motivating health workers and strengthening health workers’ telephone supervision or digital monitoring is beneficial to improving adherence.

There were still a large number of patients who were not aware of national treatment policies. In addition, consistent with other studies, our study also showed that understanding of national policy did not appear to have an impact on adherence [9]. This finding may be because among the patients who do know, some only knew what the doctor told them and did not have extensive TB knowledge. In the current study, patient need for national TB treatment policies was significantly correlated with medication adherence, and patients who listened to their doctors to take their medications on time were more likely to report stronger needs. A significant number of patients hold the belief that government should strengthen policy support, but even though the country had established some free policies for TB treatment, some items were not included in the free package, such as the cost of expensive tests and adjuvant drugs. A previous study in four Chinese provinces showed that although all smear-positive and some severe smear-negative patients received free drugs, patients still had to pay between 12 and 40% of their annual income for anti-TB treatment [47]. Participants also reported a small range and short duration of free drugs. Some patients simply could not utilize the free drugs and must spend further money elsewhere, which undoubtedly increased the financial pressure on patients. More patients also reported low reimbursement rates and high treatment costs, especially among poor and drug-resistant TB patients. The link between poverty and TB exists throughout the course of the disease, and poverty weakens TB treatment adherence [48, 49]. With strong financial security, patients were more likely to receive regular treatment and have good adherence [9]. Some participants also suggested that the state should enhance the management of TB patients and reinforce the education of those who are infectious so that they can isolate themselves. Therefore, attention should be paid to increasing financial support and strengthening the management of TB patients.

There were several limitations in our study that need to be addressed. First, self-report questionnaires were used in this study, which resulted in recall bias to some extent. Second, patients were not followed up, leading to failure to evaluate their adherence chronologically. Third, only the level of TB knowledge among patients was studied, but the ways of acquiring knowledge and the types of knowledge needed by patients were not. Fourth, the study was conducted only in one city. Due to limitations in the diversity of lifestyles and standards in different regions, adherence and factors that influence it may be different. Therefore, the results can only represent regions with the same conditions and are difficult to generalize to other regions. Finally, adherence was measured indirectly by the scale, which was less reliable than direct measurement. In a future study, regions should be expanded, and participants should be followed up to explore adherence levels and influential factors in various regions and at different treatment stages.

## Conclusion

Despite such limitations, our study can conclude that non-adherence is high in newly diagnosed TB patients. Patients who had family members who frequently supervise medication, family members who often provide spiritual encouragement, good doctor-patient relationship, more TB-related knowledge and high need for TB treatment policy support are more likely to have good medication adherence. Therefore, training and economic incentives for medical staff, health education for patients and their families at the beginning of chemotherapy, targeted increases in patient financial support, increases in patient management and psychological counselling for patients are recommended to improve patient adherence.

## Supplementary information

**Additional file 1.** Questionnaire. Questionnaire on treatment adherence among newly diagnosed tuberculosis patients in Dalian. The questionnaire was composed of socio-demographic information, adverse drug reactions, medication adherence, family support, social support and national policy support factors.

## Data Availability

The datasets used and/or analyzed during the current study are available from the corresponding author on reasonable request.

## Abbreviations

TB: Tuberculosis
MTBC: *Mycobacterium tuberculosis* complex
WHO: World Health Organization
DOT: Directly observed therapy
DOTS: Directly Observed Treatment, Short-course
OR: Odds ratio
CI: Confidence interval
