# Supplementary material for: The effects of family, society and national policy support on treatment adherence among newly diagnosed tuberculosis patients: a cross-sectional study
Source: BMC Infect Dis. 2020 Aug 24;20:623. doi: 10.1186/s12879-020-05354-3 (PMC7445902; doi:10.1186/s12879-020-05354-3)
Supplement: Supplementary file 1 — Additional file 1. Questionnaire. Questionnaire on treatment adherence among newly diagnosed tuberculosis patients in Dalian. The questionnaire was composed of socio-demographic information, adverse drug reactions, medication adherence, family support, social support and national policy support factors. [file 12879_2020_5354_MOESM1_ESM.pdf]

Questionnaire on treatment adherence of newly diagnosed tuberculosis patients in Dalian

Hello! We are the tuberculosis (TB) patients' treatment adherence research project team of Dalian Medical University, and we are investigating the treatment adherence of TB patients. The purpose of this survey is to assess the treatment adherence of newly diagnosed TB patients in Dalian and to determine the impact of family function, social support, and national policy support factors on treatment adherence. The data collected will only be used for scientific research and will not have any adverse impact on you personally. We are asking you for your generous help. We assure you that the content of your answers will be kept strictly confidential. If you do not want to participate in the study, you have full right to refuse at any time. But your honest participation will have a great contribution. So please take a few minutes to answer these questions. Thank you for your cooperation!

Do you wish to participate in the study?

……Yes, I want to participate in the study (please go to the next page)

……No, I don't want to participate

Thank you

- Questionnaire number___________________ signature _______________

Part I: Socio-demographic information and adverse drug reaction

No	Questions	Coding Categories	Code
101	Sex	1. Male 2. Female	
102	Age	_______years old	
103	Marital status	1. Unmarried<br>2. Married<br>3. Divorced or widowed	
104	What is your highest completed level of education?	1. Junior high school or below<br>2. High school or technical secondary school<br>3. College degree or above	
105	What is your monthly income (RMB)?	1. <1000<br>2. 1000-3000<br>3. 3001-5000<br>4. >5000	
106	How long does it take you to get to the medical facility from your home?	_______minutes	
107	Have you had any adverse drug reactions while taking the medicine?	1. Yes 2. No	

Part II: Adherence with medication treatment (© 2006 Donald E. Morisky)

No	Questions	Coding Categories	Code
201	Do you sometimes forget to take your TB pills?	1. Yes 2. No	
202	Over the past two weeks, were there any days when you did not take your TB medicine?	1. Yes 2. No	
203	Have you ever cut back or stopped taking your medication without telling your doctor because you felt worse when you took it?	1. Yes 2. No	
204	When you travel or leave home, do you sometimes forget to bring along your medications?	1. Yes 2. No	
205	Did you take your TB medicine yesterday?	1. Yes 2. No	
206	When you feel your condition is under control, do you sometimes stop taking your medicine?	1. Yes 2. No	
207	Taking medication everyday is a real inconvenience for some people. Do you ever feel hassled about sticking to your TB treatment plan?	1. Yes 2. No	
208	How often do you have difficulty remembering to take all your TB medication?	1. Never<br>2. Occasionally<br>3. Sometimes<br>4. Often<br>5. Always	

Part III: Family support

No	Questions	Coding Categories	Code
301	How often do your family supervise you taking the medication?	1. Never<br>2. Sometimes<br>3. Often	
302	How often can your family give you spiritual encouragement for TB treatment?	1. Never<br>2. Sometimes<br>3. Often	
303	How do your family members relate to each other?	1. Poor<br>2. General<br>3. Good	
304	How often can your family help you solve the problems in your daily life during your illness?	1. Never<br>2. Sometimes<br>3. Often	

Part IV: Society support

No	Questions	Coding Categories	Code
401	How many close friends do you have who can provide support and help?	1. 0<br>2. 1-2<br>3. ≥3	
402	How do you get along with your neighbors?	1. Poor<br>2. General<br>3. Good	
403	How do you relate to your (current or former) colleagues?	1. Poor<br>2. General<br>3. Good	
404	How is your relationship with your doctors?	1. Poor<br>2. General<br>3. Good	
405	How often will you participate in the activities of organizations (social) such as party organizations, religious organizations, trade unions, etc.?	1. Never<br>2. Sometimes<br>3. Often	
406	What do you think is the main route of transmission of TB?	1. Inhalation of TB droplet nuclei<br>2. Eat with a TB patient<br>3. Shake hands with a TB patient<br>4. Hug with a TB patient	
407	When do you think TB patients become much less infectious?	1. After taking TB antibiotics for two weeks<br>2. After two weeks of regular anti-TB treatment<br>3. When therapy is discontinued<br>4. No need for being treated	

408	What do you think are the treatment principles that TB patients must follow?	1. Early, joint<br>2. Early, joint, right amount, regular<br>3. Early, joint, regular, whole course<br>4. Early, joint, right amount, regular, whole course	
409	Do you think tuberculosis can be cured?	1. All types of TB can be cured<br>2. Most types of TB can be cured<br>3. None type of TB can be cured<br>4. Have no idea	
410	Did you know how many months of treatment can cure patients with newly active TB?	1. Four months<br>2. Six months<br>3. Eight months<br>4. Ten months	
411	What do you think are the consequences of irregular anti-TB treatment?	1. Drug resistance, making treatment difficult<br>2. Symptom decreasing<br>3. Being cured<br>4. Symptom disappearing	

Part V: National policy support

No	Questions	Coding Categories	Code
501	Do you know the national TB treatment policy?	1. Unknow<br>2. General<br>3. Know	
502	How satisfied are you with our national medical security policy for TB treatment?	1. Not too satisfaction<br>2. General satisfaction<br>3. Satisfaction	
503	How much more do you think it is necessary for the country to increase policy support for TB treatment?	1. Not too need<br>2. General need<br>3. Need	

504. What other policy support do you think the country needs to provide for the treatment of TB patients?

____

Thank you again for participating in this survey! I wish you a speedy recovery!

Investigate member:__________(signature)   The reviewer:__________(signature)

Survey date:_________________
